# Unbiased Recursive Partitioning to Stratify Patients with Acute Traumatic Spinal Cord Injuries: External Validity in an Observational Cohort Study

**DOI:** 10.1089/neu.2018.6335

**Published:** 2019-08-30

**Authors:** Nathan Evaniew, Nader Fallah, Carly S. Rivers, Vanessa K. Noonan, Charles G. Fisher, Marcel F. Dvorak, Jefferson R. Wilson, Brian K. Kwon

**Affiliations:** ^1^ Department of Orthopedics, University of British Columbia, Vancouver, British Columbia, Canada.; ^2^ Rick Hansen Institute, Vancouver, British Columbia, Canada.; ^3^ Vancouver Spine Surgery Institute, Vancouver, British Columbia, Canada.; ^4^ Division of Neurosurgery, Li Ka Shing Knowledge Institute, St. Michael's Hospital, University of Toronto, Toronto, Ontario, Canada.; ^5^ International Collaboration on Repair Discoveries (ICORD), University of British Columbia, Vancouver, British Columbia, Canada.

**Keywords:** bias, clinical research, spinal cord injury, statistical error, stratification

## Abstract

Clinical trials of novel therapies for acute spinal cord injury (SCI) are challenging because variability in spontaneous neurologic recovery can make discerning actual treatment effects difficult. Unbiased Recursive Partitioning regression with Conditional Inference Trees (URP-CTREE) is a novel approach developed through analyses of a large European SCI database (European Multicenter Study about Spinal Cord Injury). URP-CTREE uses early neurologic impairment to predict achieved motor recovery, with potential to optimize clinical trial design by optimizing patient stratification and decreasing sample sizes. We performed external validation to determine how well a previously reported URP-CTREE model stratified patients into distinct homogeneous subgroups and predicted subsequent neurologic recovery in an independent cohort. We included patients with acute cervical SCI level C4–C6 from a prospective registry at a quaternary care center from 2004–2018 (*n* = 101) and applied the URP-CTREE model and evaluated Upper Extremity Motor Score (UEMS) recovery, considered correctly predicted when final UEMS scores were within a pre-specified threshold of 9 points from median; sensitivity analyses evaluated the effect of timing of baseline neurological examination. We included 101 patients, whose mean times from injury baseline and follow-up examinations were 6.1 days (standard deviation [SD] 17) and 235.0 days (SD 71), respectively. Median UEMS recovery was 7 points (interquartile range 2–12). One of the predictor variables was not statistically significant in our sample; one group did not fit progressively improving UEMS scores, and three of five groups had medians that were not significantly different from adjacent groups. Overall accuracy was 75%, but varied from 82% among participants whose examinations occurred at <12 h, to 64% at 12–24 h, and 58% at >24 h. A previous URP-CTREE model had limited ability to stratify an independent into homogeneous subgroups. Overall accuracy was promising, but may be sensitive to timing of baseline neurological examinations. Further evaluation of external validity in incomplete injuries, influence of timing of baseline examinations, and investigation of additional stratification strategies is warranted.

## Introduction

Clinical trials of novel therapies for acute traumatic spinal cord injuries (SCIs) are extremely challenging because variability in spontaneous neurologic recovery can make discerning actual treatment effects difficult.^1^ Differences in neurological level of injury, severity of neurological impairment, spinal column stability, age, timing of enrollment, or medical comorbidities are each associated with important differences in prognosis, and researchers must implement methodological safeguards to avoid spurious or misleading results.^1–4^ One such safeguard is stratification, which can be used in randomized controlled trials to ensure balanced groups while maintaining efficient enrollment.^5–7^

Unbiased Recursive Partitioning regression with Conditional Inference Trees (URP-CTREE) is a novel approach to stratification that was developed through analyses of the European Multicenter study about Spinal Cord Injury (EMSCI) database.^8–11^ URP-CTREE is a statistical technique that uses early neurologic impairment to predict how much motor recovery individual patients will achieve, and sequentially partitions initially heterogeneous groups into increasingly more homogenous groups.^12^ Tanadini and colleagues^8^ applied URP-CTREE to an EMSCI sample of 159 patients with complete acute traumatic cervical spinal cord injuries and evaluated its prediction accuracy for upper extremity motor score (UEMS) scores at 6 months post-injury. Their results suggested that URP-CTREE might optimize future clinical trials by providing a data-driven approach to early patient stratification.

While the EMSCI URP-CTREE is a potentially promising tool for acute SCI clinical trials, the system has not been externally validated on an independent dataset. Such an external validation would be helpful for understanding how the URP-CTREE system might perform in a subsequent clinical trial with an independent cohort of patients. Therefore, we performed an external validation study to determine how well a previously reported URP-CTREE model stratified patients into distinct homogeneous subgroups and predicted subsequent neurologic recovery when applied to an independent cohort of patients' data from an ongoing prospective observational study of patients with acute traumatic cervical SCIs.

## Methods

### Study design

We performed a retrospective analysis of data that were prospectively collected at the Vancouver General Hospital as part of the Rick Hansen Spinal Cord Injury Registry (RHSCIR) in Canada. RHSCIR is an ongoing multi-center prospective observational study of patients with acute traumatic spinal cord injuries, and the Vancouver General Hospital is a major academic quaternary care referral center that is part of the RHSCIR network. We obtained local Research Ethics Board approval prior to enrolling patients, collecting data, and performing this study. Further descriptions of the RHSCIR data elements, procedures, governance structure, and privacy and confidentiality framework have been previously reported.^13^

### Patient sample

We included all patients enrolled from May 2004 to February 2018 that met the eligibility criteria reported by Tanadini and colleagues of presenting with complete American Spinal Injury Association (ASIA) Impairment Scale (AIS) A acute traumatic cervical spinal cord injuries with baseline motor levels at C4 to C6 on the right side of their bodies.^8^ We excluded patients with incomplete baseline or follow-up neurological examinations. We also excluded patients whose baseline neurological examinations were recorded more than two weeks after their injuries, or whose discharge neurological examinations occurred at less than 5 months post-injury.

### Data sources

All data were collected by trained research personnel and entered into the standardized local RHSCIR database before being exported to the RHSCIR national office for centralized quality checks. We extracted age, motor level (right body side), bilateral sensory and motor scores, and zones of partial preservation for analysis as baseline predictors.^8^ We also extracted patients' age at injury, mechanism of injury, Glasgow Coma Scale (GCS) score, Injury Severity Score (ISS), and data on whether they were treated with surgery as descriptors of our cohort. Missing or ambiguous data were reconciled with local research coordinators, hospital health records, and medical chart abstraction whenever possible.

Neurological examinations were performed according to the International Standards for Neurological Classification of Spinal Cord Injury (ISNCSCI) by trained physicians, nurse practitioners, or physiotherapists.^14^ ISNCSCI total motor scores (TMS) can range from 0 (absent motor function) to 100 (intact motor function) and comprise UEMS (range 0–50) and lower extremity motor scores (range 0–50). Zones of partial preservation (ZPP) refer to segments caudal to the ISNCSCI neurological levels where there is partial preservation motor or sensory function. ISNCSCI records were processed through a validated computerized algorithm that maintained consistency and high quality.^15^ We considered baseline motor scores to be those obtained on admission to acute care and final motor scores to be those obtained at the time of discharge to the community from acute care or inpatient rehabilitation.

### Statistical analysis

URP-CTREE recursively partitions samples into binary tree configurations that maximize goodness of fit according to two-sample linear statistics.^12^ It produces models with branch points that occur sequentially at predictors whose univariate associations to the outcome of interest are greatest, and whose splits at each predictor are most efficient in comparison to all other potential splits. Branching continues until the remaining predictors do not have statistically significant univariate associations. In this study, we applied the existing URP-CTREE model reported by Tanadini and colleagues and partitioned our sample according to the predictors and splits derived from EMSCI as if they were being used to stratify enrollment in a clinical trial.^8^

We report discrete variables as counts or proportions, normally distributed continuous variables as means with standard deviations (SDs), and skewed continuous variables as medians with interquartile ranges (IQRs). We used parametric tests for data with normal distributions and non-parametric tests for data without normal distributions. We tested univariate associations with the Pearson correlation coefficient and differences between medians with the Mann-Whitney U test. We considered recovery to be correctly predicted when final UEMS scores were within a pre-specified threshold of 9 points from the median in each group, because that threshold was recently used in the sample size calculation of a current definitive randomized controlled trial of a neuroprotective agent in patients with acute traumatic complete and incomplete cervical SCI.^16^ We also performed a sensitivity analysis with a threshold of 5 points because studies of complete SCI might implement a narrower change. Patients with missing data were excluded from each analysis and imputations were not performed. All tests of significance were two-tailed and *p* values of less than 0.05 were considered significant except when Bonferroni corrections were applied to adjust for multiple testing. Boxplots depict medians, the first and third quartiles (Q1 and Q3, respectively), and outliers more than 1.5 IQRs beyond Q1 and Q3. Probability density plots present the probability of an enrolled patients being partitioned into each node. We performed our analyses with Excel 2011 (Microsoft Corp., Redmond, WA), and IBM SPSS Version 21, 2012 (SPSS Inc., Chicago IL).

## Results

### Patient sample

**FIG. 1.**
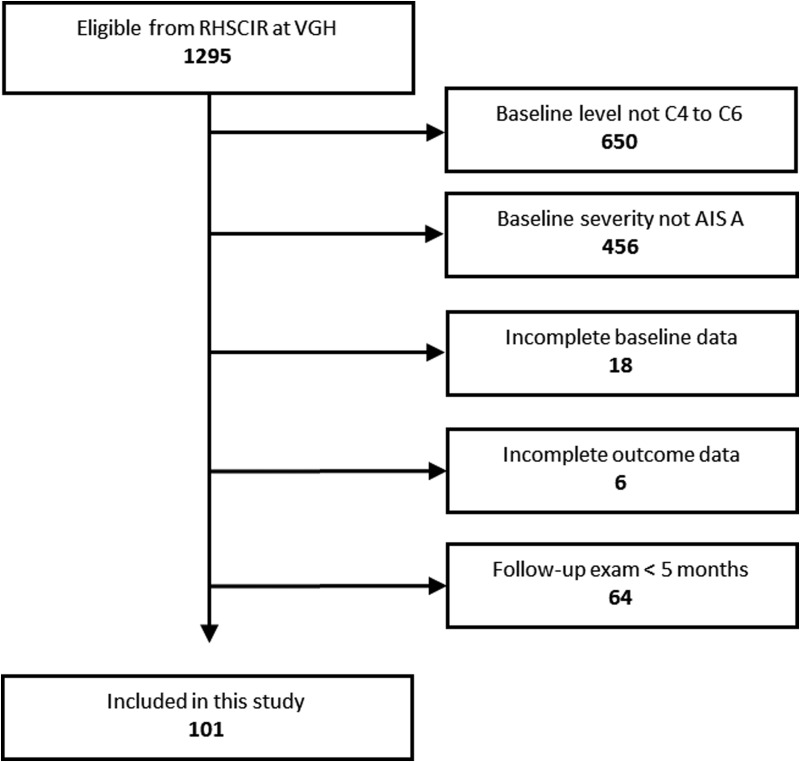
Flow of patient inclusion and exclusion. RHSCIR, Rick Hansen Spinal Cord Injury Registry; VGH:, Vancouver General Hospital; AIS: American Spinal Injury Association Impairment Scale.

Of 1295 patients with acute traumatic SCIs who consented to enrollment and were subsequently discharged to the community from acute care or inpatient rehabilitation, we excluded 650 because their baseline motors levels were not at C4 to C6, 456 because their baseline injury severity was not AIS A, 18 because they had incomplete baseline data, 64 because they did not have a discharge neurological examination at ≥5 months post-injury, and six because they had incomplete outcome data (Fig. 1). In total, we included 101 cervical A patients whose mean age was 43 (SD 18), GCS was 14 (SD 3), and ISS was 29 (SD 11; Table 1). Baseline injury level was C4 in 38 (38%), C5 in 52 (51%), and C6 in 11 (11%). Eight-four (83%) patients were male and 93 (92%) were treated with surgery. Mean time from injury to baseline neurological examination was 6 days (SD 17) and mean time from injury to final neurological examination was 235 days (SD 71). Median UEMS recovery was 7 points (IQR 2 to 12).

**Table 1.** Patient Sample: Baseline Characteristics

*Baseline characteristics (*n* = 101)*	
Age: mean (SD)	43 (18)
Male sex: *n* (%)	84 (83)
Mechanism of injury: *n* (%)	39
Motor vehicle accident	19
Sport	36
Fall	5
Assault	2
Other	
Baseline neurological injury level: *n* (%)	
C4	38 (38)
C5	52 (51)
C6	11 (11)
Glasgow Coma Scale: mean (SD)	14 (3)
Injury Severity Score: mean (SD)	29 (11)
Treated with surgery: *n* (%)	93 (92)
Time from injury to baseline examination, days: mean (SD)	6 (17)
Time from injury to final examination, days: mean (SD)	235 (71)
Baseline UEMS: median (IQR)	10 (6 to 20)
Final UEMS: median (IQR)	20 (15 to 28)
UEMS recovery: median (IQR)	7 (IQR 2 to 12)

SD, standard deviation; UEMS, Upper Extremity Motor Score, IQR, interquartile range.

### Application of the EMSCI URP-CTREE model to our sample

**FIG. 2.**
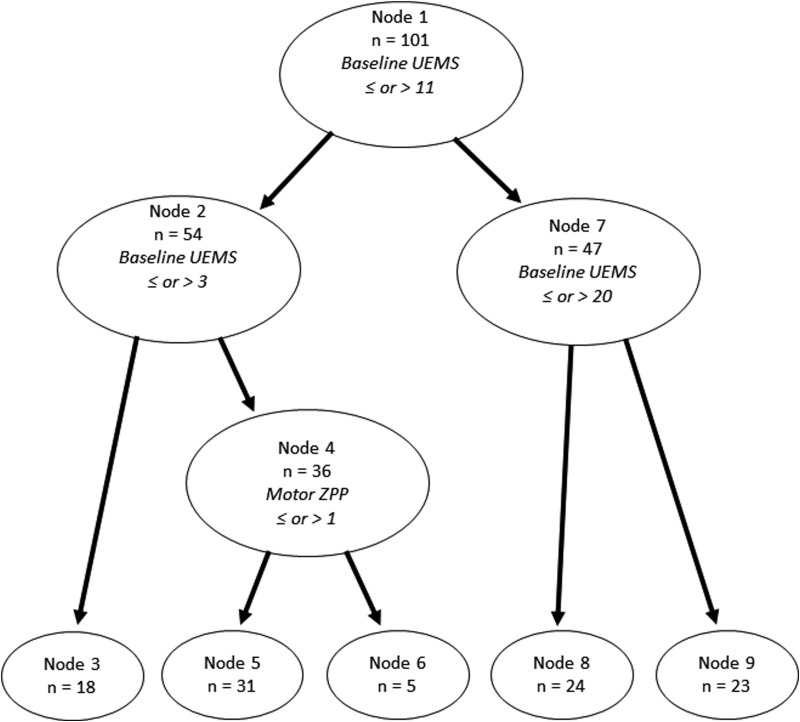
Application of the Unbiased Recursive Partitioning regression with Conditional Inference Trees (URP–CTREE) model for patient stratification from the European Multicenter study about Spinal Cord Injury (EMSCI) to our patient sample. UEMS, Upper Extremity Motor Score; ZPP, zone of partial preservation.

**FIG. 3.**
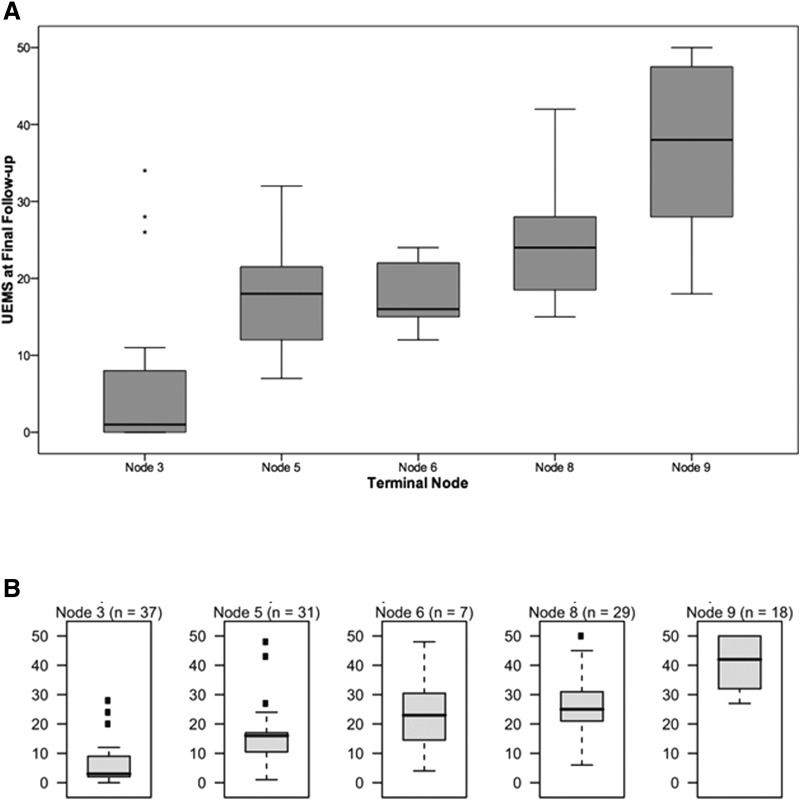
Distributions of Upper Extremity Motor Score (UEMS) among terminal nodes at final follow–up in the current study **(A),** and in the original report by Tanadini and colleagues.^8^ **(B).** Panel (B) reproduced in part from Tanadini and colleagues,^8^ with permission.

We applied the previously reported EMSCI URP-CTREE model, which partitioned our cohort into five stratified groups of predicted UEMS recovery (Fig. 2). The first partition occurred according to baseline UEMS scores of less than or equal to 11 versus greater than 11 at Node 1. These subgroups were then further partitioned according to baseline UEMS scores of less than or equal to three versus greater than three at Node 2, and less than or equal to 20 versus greater than 20 at Node 3. Node 4, further partitioned a subset of patients from Node 2 according to baseline motor ZPP less than or equal to one level versus greater than one level. The model yielded five terminal nodes, with sizes that varied from five to 31 patients and sequential median predicted UEMS scores of 1 (IQR 0 to 8), 18 (IQR 12 to 22), 16 (IQR 15 to 22), 24 (IQR 19 to 28), and 38 (IQR 28 to 48; Fig. 3A). For comparison, the original model from Tanadini and colleagues from EMSCI for the same endpoint of total UEMS at 6 months among patients with cervical complete (AIS A) injuries (*n* = 122) is shown in Figure 3B.

**FIG. 4.**
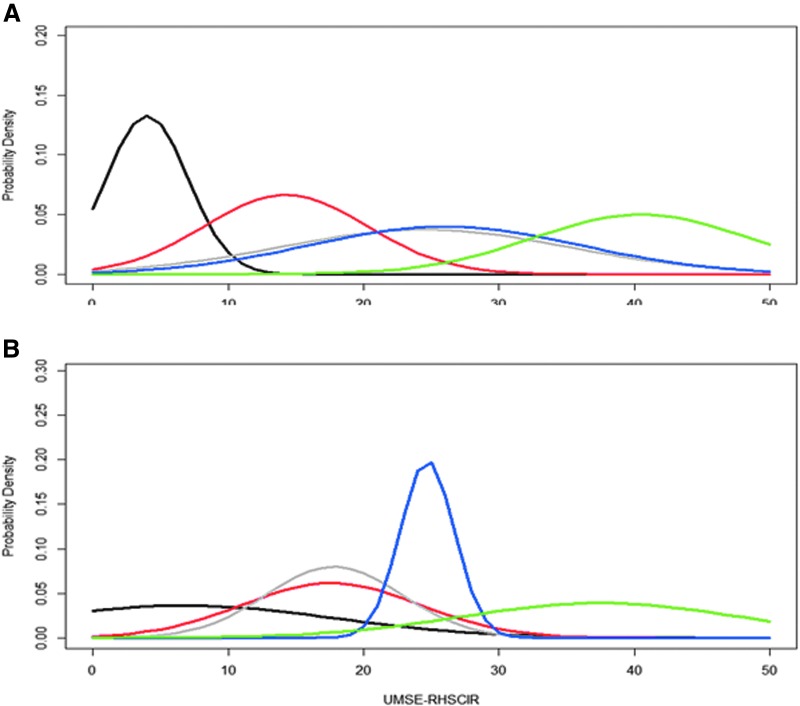
Probability density plot for five different Upper Extremity Motor Scores (UEMS) at follow–up in **(A)** European Multicenter study about Spinal Cord Injury (EMSCI) dataset and **(B)** Rick Hansen Spinal Cord Injury Registry (RHSCIR) dataset. Black is for Node 3, red for Node 5, gray for Node 6, blue for Node 8 and green for Node 9. Color image is available online.

We present probability density plots for the nodes from each dataset in Figure 4. The EMSCI model appeared to have weak differentiation between Nodes 6 and 8, indicated by similar distributions of UEMS scores at final follow-up, and the RHSCIR model from our current study did not reproduce that result. In Figure 4B, there is greater overlap of the distributions from each node, with Nodes 5 and 6 overlaid. These results indicate that the model has limited ability to stratify patients into distinct homogeneous groups.

We found that one of the four predictor variables failed to reach statistical significance in our independent sample (Node 4: Motor ZPP, *n* = 36; Table 2), one of the stratified groups did not fit a sequence of progressively improving UEMS scores (Node 6), and three of the five stratified groups had medians that were not statistically significantly different from their adjacent medians (Table 3). In a sensitivity analysis in which we included only those patients whose time from injury to baseline neurological examination was less than 24 h (*n* = 63, mean time = 10 h [SD 6], median UEMS recovery was 6 points [IQR 2 to 12]), one of the predictor nodes was not statistically significant, and four of five medians were not significantly different from their adjacent medians (Appendix 1). There were no statistically significant findings when we included only those patients whose time from injury to baseline neurological examination was less than 12 h (*n* = 38, mean time = 6.1 h [SD 0.2], median UEMS recovery 7.5 points [IQR 3 to 13]; Appendix 2).

**Table 2.** Univariate Associations with Predictor Variables at Each Node

*Predictor*	*Correlation*	p *value^1^*
Node 1: Baseline UEMS (*n* = 101)	0.837	< 0.01
Node 2: Baseline UEMS (*n* = 54)	0.609	< 0.01
Node 4: Motor ZPP (*n* = 36)	0.131	0.11
Node 7: Baseline UEMS (*n* = 47)	0.803	< 0.01

^1^ Pearson correlation coefficient.

The *p* values have been adjusted for multiple comparisons using a Bonferroni correction and <0.01 was considered significant.

UEMS, Upper Extremity Motor Score; ZPP, zone of partial preservation.

**Table 3.** Comparisons of Medians at Adjacent Terminal Nodes

*Comparison*	p *value^1^*
Node 3 vs. Node 5	< 0.01
Node 5 vs. Node 6	0.08
Node 6 vs. Node 8	0.20
Node 8 vs. Node 9	< 0.01

^1^ Mann–Whitney U test.

The *p* values have been adjusted for multiple comparisons using a Bonferroni correction and <0.01 was considered significant.

Overall, the model accurately predicted final motor recovery within 9 points of the median at each node in 76 patients (75%). Prediction accuracy among participants whose examinations occurred at less than 12 h was 82%, 12 to 24 h was 64%, and greater than 24 h was 58%. In a sensitivity analysis with a threshold of 5 points rather than 9, overall accuracy was 49%, at less than 12 h was 55%, at 12 to 24 h was 64%, and greater than 24 h was 32%.

## Discussion

We performed an external validation study to determine how well a previously reported URP-CTREE model stratified patients into distinct homogeneous subgroups and predicted subsequent neurologic recovery when applied to an independent cohort. We found that the model had limited ability to stratify patients into distinct homogeneous subgroups and that overall accuracy for predicting final motor recovery was reasonably promising but may be sensitive to timing of baseline neurological examinations.

### Limitations and strengths

Our study included data from only one site in the RHSCIR network, which raises the possibility that our findings may reflect a small sample size or have limited applicability to other centers and healthcare systems. However, our center is known to have the highest volume of acute traumatic spinal cord injury admissions in Canada,^17^ our sample size was comparable to that described by Tanadini and colleagues in their index report on URP-CTREE,^8^ and the patients in our study had similar epidemiology and underwent similar management to patients at other specialized centers nationally and internationally.^18,19^ Further, the patients in our sample experienced a magnitude of motor recovery comparable to that reported elsewhere for cervical complete injuries.^20,21^

We limited our analysis to patients with cervical AIS A injuries in order to specifically evaluate the external validity of the EMSCI URP-CTREE model in this population; therefore, our results do not directly inform about the external validity of URP-CTREE for patients with incomplete injuries. Nonetheless, our study has some indirect application to patients with injuries at other anatomical levels and with varying severity. Whereas challenges of predicting neurological outcomes and stratifying patients efficiently are likely to be even greater when baseline heterogeneity is increased, it was critically important that URP-CTREE be externally validated initially under ideal circumstances. Our sample of C4–C6 AIS A patients closely resembled the sample from EMSCI and likely provided the most favorable cohort in which to evaluate the EMSCI model. In an analysis that compared data from 122 EMSCI patients with AIS B and C injuries to data from 83 patients who were enrolled in a randomized trial of GM-1 ganglioside (Sygen), Tanadini and colleagues reported similar distributions of 6-month UEMS scores using a URP-CTREE model with nodes for baseline UEMS and light touch sensory scores.^9,22^ Reproduction of these findings in another cohort of patients with incomplete traumatic spinal cord injuries such as RHSCIR remains an important knowledge gap, and further research is warranted before those results are applied to the design of trials for novel interventions.

The EMSCI model was created using data from participants whose baseline examinations occurred at a mean of 8.1 days (SD 4.7) post-injury. Our cohort had earlier baseline examinations than the majority of patients in the EMSCI database, but they were still later than the requirements of typical acute clinical trials (i.e., within 12–24 h post–injury). Delayed neurological examinations may miss substantial early neurological recovery and introduce considerable bias, and our finding of worsening prediction accuracy with increasing intervals from injury to baseline examination suggests that this bias could occur according to a dose–response relationship.^2,20,23^ However, our findings of similar UEMS among patients examined at less than 12 h and 24 h post–injury in comparison to our total cohort UEMS recovery do not support this argument. Our specific finding of worse accuracy with delayed examinations seems counterintuitive because we expected early examinations to yield less accuracy in the context of an EMSCI model based on delayed examinations, but our study nonetheless highlights that the URP–CTREE approach needs further refinement and assessment before being generally implemented in trials. The interpretation of results from studies with delayed baseline neurological exams may require caution, and data from studies with delayed neurological examinations may have limited applicability to the design of trials that require early enrolment.

We implemented a threshold of 9 points to detect clinically important changes in UEMS prediction accuracy because it had been used elsewhere, but the minimum clinically important differences (MCIDs) in UEMS and TMS remain controversial in the literature. Some investigators have estimated MCIDs to be larger than 9 points, while others argue that smaller changes might be very meaningful if they occur at myotomes that impact quality of life.^1,7^ In an analysis of 600 patients with prospectively collected ISNCSCI data from a single rehabilitation hospital in Italy, Scivoletto and colleagues applied several distribution–based approaches to estimate clinical significance and found that 5– and 11– point motor score changes were associated with clinically significant improvements of 0.2 and 0.5 SD units, respectively.^24^ They suggested that the proportion of subjects who achieve clinically significant improvements should be a preferred outcome when comparing the effects of interventions. It is also plausible that MCIDs for SCI might vary with baseline level, severity, and even chronicity.^25^ Our sensitivity analysis showed decreased accuracy when we selected a smaller threshold, and we would have undoubtedly found increased accuracy if our threshold was greater. In general, it is difficult to say exactly how close to the original URP–CTREE findings the results of this external validation study would need to be in order to confirm the robustness of the initial prediction. Ultimately, researchers looking to utilize this URP–CTREE for clinical trial purposes will have to consider this in the context of their specific research question and study design.

We excluded patients whose discharge neurological exams occurred at less than 5 months post–injury in order to generate a cohort with at least approximately 6 months of follow–up. We excluded patients whose discharge neurological exams occurred at less than 5 months post– injury in order to generate a cohort with at least approximately 6 months of follow–up. This allowed us to replicate the follow–up for the primary UEMS analyses of Tanadini and colleagues, which also was 6 months post–injury. However, we did not replicate their secondary analysis that was based on 12–month data because our RHSCIR cohort does not routinely include 12–month motor score follow–up. That secondary analysis partitioned patients into two groups on the basis of whether or not they were predicted to achieve neurological improvements by two or more motor levels. We were also unable to directly compare the distributions of UEMS for each node in our model to their original model because we did not have access to their original data. Nonetheless, the medians and distributions that we report in Figure 3A are broadly similar to those shown from Tanadini and colleague in Figure 3B.

This study did not control for potential confounding due to timing of surgery or to timing of neurological examinations relative to surgery, and it is possible that these omissions could have influenced our results. For example, patients who underwent earlier surgery and patients whose baseline examinations occurred before surgery may have been more likely to experience greater apparent neurological recovery than those with delayed examinations. Although a recent observational study from RHSCIR found a beneficial effect of earlier surgery among patients with incomplete injuries but not complete injuries,^23^ further research is warranted to explore potential bias due to variations in baseline neurological examination timing, including in relation to timing of surgery.

We were also unable to explore or control for potential differences in factors that were not part of the EMSCI URP–CTREE model but may have influenced neurological recovery, such as differences in baseline characteristics and treatments. The report by Tanadini and colleagues^8^ did not include a detailed description of these factors and we did not have access to the EMSCI database in order to evaluate them directly. Further work is warranted to explore the feasibility of potentially merging the RHSCIR database, EMSCI database, and other sources in order to bridge this critical knowledge gap.

### Relation to previous literature

This external validation study supports a growing body of literature that has attempted to accurately and reliably predict outcomes after traumatic SCI. Recent reports have highlighted the importance of understanding baseline clinical heterogeneity, and have illustrated how failures to acknowledge differences among participants can undermine trials designed to evaluate promising therapies. For example, an analysis of 836 patients from the RHSCIR database found that clinically meaningful motor score recovery could be predictably related to a joint distribution of the neurological level of injury and injury severity, but it failed to identify a statistical difference in prognosis between high (C1–C4) and low (C4–C6) AIS A.^2^ Our study suggests that the use of early (< 2 weeks post–SCI) motor scores and the zone of partial preservation to predict 6–month motor scores has definite merit, but the precision with which these early neurologic findings separated patients into distinct subgroups was rather modest.

URP–CTREE is one of many statistical techniques that can be applied to datasets in order to predict outcomes. More common alternatives include conventional linear or logistic regression, generalized linear modeling, other types of machine learning techniques, and other decision tree–based methods. To date, URP–CTREE has seen only limited implementation in the medical literature and even less so in the field of spinal cord injury. Velstra and colleagues^10,11^ applied URP–CTREE to Graded and Redefined Assessment of Strength, Sensibility, and Prehension data from EMSCI in order to predict upper limb function and self–care after acute SCI, but we are unaware of any other investigations of external validity in cervical complete patients. The practical utility of URP–CTREE in comparison to established methods that are more familiar to researchers and clinicians remains unclear, although it would seemingly be appropriate to at least try implementing it in a prospective clinical trial in which early subject recruitment was undertaken (thus requiring early baseline neurologic assessment) to evaluate the utility of this approach for predicting outcome and potentially reducing sample size.

### Implications

The need to predict neurologic outcome with better accuracy is a translational imperative for the SCI field to enable the evaluation of acute clinical interventions. As such, initiatives such as the URP–CTREE are extremely valuable for the field. Improved prediction may be achieved by combining such clinical features with objective magnetic resonance imaging biomarkers and/or neurochemical biomarkers from cerebrospinal fluid or blood.^7,26,27^ While the use of early motor scores to predict later recovery is conceptually appealing for clinicians, our application of this approach to an independent dataset of complete SCI subjects did not reveal the ability to clearly discern different motor score outcomes. Further study is therefore warranted to establish the parameters by which early motor scores may be used to substratify patients who are enrolled into clinical trials of novel therapeutics. The role of “timing of assessment” is also something that requires further study, given that clinical trials of novel therapeutics in the acute setting often require intervention to be started within 12–24 h (i.e., within a very acute time frame in the first 2 weeks post–injury). The variation in spontaneous recovery with such an early baseline examination may certainly alter how well such early assessments can be used to predict outcome.

While the recursive partitioning concept was developed to assist in the stratification of patients for acute clinical trials, it is acknowledged that the application could be much broader. The more accurate prediction of long–term neurological outcomes is not only directly relevant to patients who suffer these injuries but also to their caregivers and the broad group of healthcare providers and healthcare administrators who are involved in their medical and rehabilitative treatment. A method for more accurately predicting motor score recovery at the outset would also help to establish realistic rehabilitation goals and guide subsequent physical and occupational therapy.

## Conclusions

A previously reported URP–CTREE model had limited ability to stratify an independent cohort of patients with acute cervical AIS A injury into distinct homogeneous subgroups. Overall accuracy for predicting final motor recovery was reasonably promising, but may be sensitive to the timing of baseline neurological examinations. Further research is warranted to evaluate the external validity of URP–CTREE among patients with incomplete injuries and to investigate additional strategies for accurately stratifying patients with acute SCI.

## Figures and Tables

**APPENDIX 1 FIG. A1.**
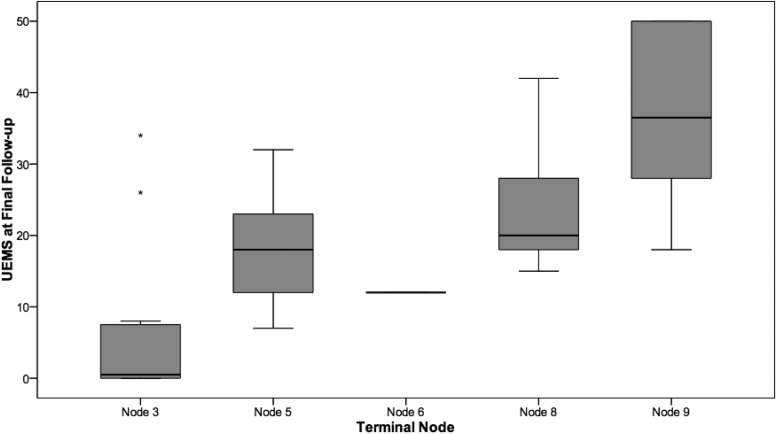
Distributions of Upper Extremity Motor Score (UEMS) among terminal nodes at final follow–up.

**APPENDIX 2 FIG. A1.**
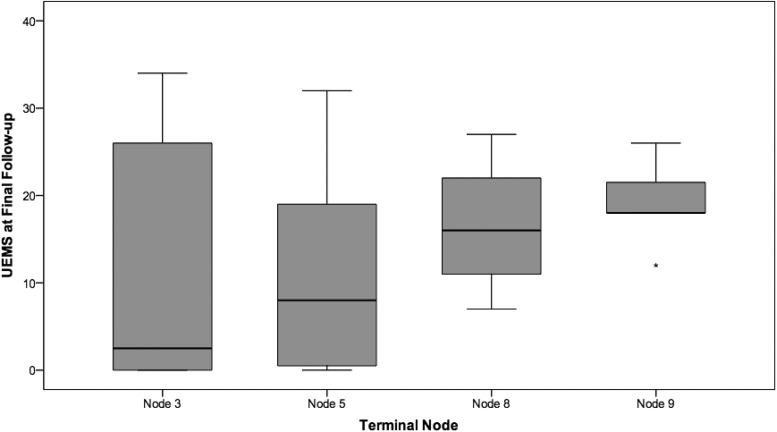
Distributions of Upper Extremity Motor Score (UEMS) among terminal nodes at final follow–up.

## Appendix 1

Sensitivity analyses in which only those patients whose time from injury to baseline neurological examination was less than 24 h were included (*n* = 63; mean time to examination = 10 h, SD 6; median Upper Extremity Motor Score recovery 6 points, interquartile range 2 to 12).

**Appendix 1 Table A1.** Univariate Associations with Predictor Variables at Each Node

*Predictor*	*Correlation*	p *value^1^*
Node 1: Baseline UEMS (*n* = 63)	0.819	< 0.01
Node 2: Baseline UEMS (*n* = 34)	0.584	< 0.01
Node 4: Motor ZPP (*n* = 22)	0.184	0.10
Node 7: Baseline UEMS (*n* = 29)	0.830	< 0.01

^1^ Pearson Correlation Coefficient; *p* values have been adjusted for multiple comparisons using a Bonferroni correction.

UEMS, Upper Extremity Motor Score, ZPP, zone of partial preservation.

**Appendix 1 Table A2.** Comparisons of Medians at Terminal Nodes

*Comparison*	p *value^1^*
Node 3 vs Node 5	< 0.01
Node 5 vs Node 6	0.25
Node 6 vs Node 8	0.25
Node 8 vs Node 9	0.03

^1^ Mann–Whitney U test; *p* values have been adjusted for multiple comparisons using a Bonferroni correction.

## Appendix 2

Sensitivity analyses in which only those patients whose time from injury to baseline neurological examination was less than 12 h were included (*n* = 38, mean time = 6.1 h, SD 0.2; median Upper Extremity Motor Score recovery 7.5 points, interquartile range 3 to 13).

**Appendix 2 Table A1.** Univariate Associations with Predictor Variables at Each Node

*Predictor*	*Correlation*	p *value^1^*
Node 1: Baseline UEMS (*n* = 38)	0.26	0.04
Node 2: Baseline UEMS (*n* = 17)	0.05	0.21
Node 4: Motor ZPP (*n* = 11)^2^	–	–
Node 7: Baseline UEMS (*n* = 21)	0.05	0.21

^1^ Pearson Correlation Coefficient. p–values have been adjusted for multiple comparisons using a Bonferroni correction.

^2^ Patients were not be further partitioned by ZPP because there were no patients in Node 6.

UEMS, Upper Extremity Motor Score; ZPP, zone of partial preservation.

**Appendix 2 Table A2.** Comparisons of Medians at Terminal Nodes

*Comparison*	p *value^1^*
Node 3 vs Node 5	0.31
Node 5 vs Node 6^2^	–
Node 6 vs Node 8	–
Node 8 vs Node 9	0.50

^1^ Mann–Whitney U test.

^2^ There were no patients in Node 6.
